# Plasmonics Meets Perovskite Photovoltaics: Innovations and Challenges in Boosting Efficiency

**DOI:** 10.3390/molecules29215091

**Published:** 2024-10-28

**Authors:** Chen Wang, Xiaodan Wang, Bin Luo, Xiaohao Shi, Xiangqian Shen

**Affiliations:** 1 Xinjiang Key Laboratory of Solid State Physics and Devices, School of Physical Science and Technology, Xinjiang University, Urumqi 830046, China; w18336964902@163.com (C.W.); 107552303626@stu.xju.edu.cn (B.L.); 107552303638@stu.xju.edu.cn (X.S.); 2 Department Interface Design, Helmholtz-Zentrum Berlin für Materialien und Energie GmbH (HZB), Albert-Einstein-Str. 15, 12489 Berlin, Germany; xiaodan.wang@helmholtz-berlin.de; 3 State Key Laboratory of Metal Matrix Composites, Shanghai Jiao Tong University, Shanghai 200240, China

**Keywords:** perovskite solar cells, plasmonic nanoparticles, interface engineering, light harvesting, photovoltaic performance

## Abstract

Perovskite solar cells (PSCs) have garnered immense attention in recent years due to their outstanding optoelectronic properties and cost-effective fabrication methods, establishing them as promising candidates for next-generation photovoltaic technologies. Among the diverse strategies aimed at enhancing the power conversion efficiency (PCE) of PSCs, the incorporation of plasmonic nanoparticles has emerged as a pioneering approach. This review summarizes the latest research advancements in the utilization of plasmonic nanoparticles to enhance the performance of PSCs. We delve into the fundamental principles of plasmonic resonance and its interaction with perovskite materials, highlighting how localized surface plasmons can effectively broaden light absorption, facilitate hot-electron transfer (HET), and optimize charge separation dynamics. Recent strategies, including the design of tailored metal nanoparticles (MNPs), gratings, and hybrid plasmonic–photonic architectures, are critically evaluated for their efficacy in enhancing light trapping, increasing photocurrent, and mitigating charge recombination. Additionally, this review addresses the challenges associated with the integration of plasmonic elements into PSCs, including issues of scalability, compatibility, and cost-effectiveness. Finally, the review provides insights into future research directions aimed at advancing the field, thereby paving the way for next-generation, high-performance perovskite-based photovoltaic technologies.

## 1. Introduction

With the ongoing consumption of global energy and the escalating issues of environmental, the demand for green, clean, and renewable energy sources has become increasingly urgent. Among the various green energy options, solar energy is particularly notable due to their environmentally friendly, convenient utilization, and abundant availability [1,2,3]. Solar cells, which are pivotal devices for converting solar energy into electrical energy, play a crucial role in advancing the adoption of clean energy [4,5,6,7,8,9,10,11]. Perovskite Solar Cells (PSCs), as a pioneering class within thin-film photovoltaics, have garnered significant attention in recent years due to their remarkable progress in power conversion efficiency (PCE) [12,13,14,15,16,17,18,19]. Since their inception in 2009 with an initial PCE of 3.8%, PSCs have witnessed a meteoric rise, achieving a certified efficiency of over 26% in just a few years, surpassing many other emerging solar cell technologies [20,21,22,23,24,25,26,27,28,29,30,31,32]. This remarkable improvement can be attributed to their exceptional optoelectronic properties, such as high absorption coefficients, tunable bandgaps, and long carrier diffusion lengths [33,34,35]. However, despite these advantages, PSCs still face challenges related to their optical and electrical performance, particularly in terms of light absorption and charge transport, which limit their overall efficiency and stability [36,37,38,39,40,41]. To this end, researchers have explored diverse strategies aimed at optimizing PSC performance, from improving material purity and stability to innovating device architectures and interfacial engineering [42,43,44].

**Figure 1:**
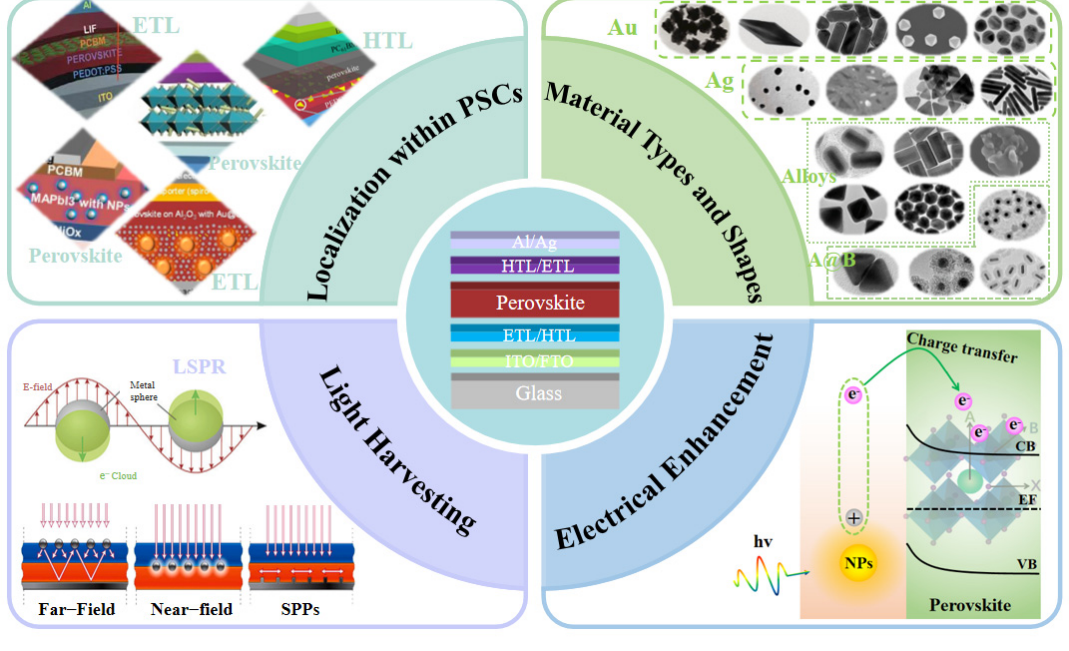
The strategies and enhancement mechanisms of plasmonic nanostructures in PSCs. Reproduced with permission from Ref. [69]. Copyright 2003, American Chemical Society. Reproduced with permission from Ref. [70]. Copyright 2010, Nature.

One of the most promising avenues in this endeavor is the integration of plasmonic nanostructures into PSC designs. Plasmonic nanostructures represent an innovative class of materials that harness the unique optical properties of surface plasmons, the collective oscillations of electrons at the interface between a metal and a dielectric material [45,46,47]. These nanostructures can effectively manipulate light at the nanoscale, enabling precise control over light absorption, scattering, and confinement. The most commonly used materials for plasmonic nanostructures are metal nanoparticles (MNPs) with a high free electron density, particularly Au, Ag, and Al, due to their strong ability to support surface plasmons [48,49,50]. In addition to these, there are several other plasmonic materials, including metallic materials such as Mg, In, Na, and K; carbon-based materials like carbon nanotubes and graphene; metal alloy materials; semiconductor materials like indium–tin oxide (ITO), aluminum–zinc oxide (AZO), gallium–zinc oxide (GZO), GaAs, GaN, and GaP; as well as composite materials [51,52,53,54,55,56,57]. By tuning the size, shape, and composition of plasmonic nanostructures, researchers can engineer their optical resonances to match specific wavelengths, thus enhancing the interaction between light and matter in a desired manner [58,59,60,61,62,63,64,65]. In the context of PSCs, plasmonic nanostructures offer a promising solution by enhancing light absorption in the perovskite layer and facilitating efficient charge extraction. These structures can be categorized according to their placement within different layers, such as electron transport layer (ETL) [66], hole transport layer (HTL) [67], and perovskite active layers [68]. Figure 1 illustrates the strategies and enhancement mechanisms of plasmonic nanostructures in PSCs. Specifically, when integrated into PSCs, plasmonic nanoparticles, nanowires, or metasurfaces can concentrate and redirect incident sunlight into the active layer. The localized surface plasmon resonance (LSPR) of these nanostructures leads to significant absorption enhancements, particularly in the near-infrared and visible regions where the perovskite material might otherwise have limited absorption. This enhancement increases the overall photocurrent generation, a key parameter determining the efficiency of solar cells. Furthermore, plasmonic nanostructures can act as scattering centers, prolonging the optical path of light within the perovskite film, allowing for multiple absorption events. This scattering effect also contributes to a broader absorption spectrum and improved light harvesting efficiency. In addition to these optical enhancements, plasmonic nanostructures have also demonstrated their capability in improving the electrical performance of PSCs. For one thing, plasmon decay can generate hot electrons with energies exceeding the bandgap of the perovskite material. These hot electrons can be injected into the perovskite, contributing to the photocurrent and potentially bypassing some of the loss mechanisms associated with thermalization. For another, plasmonic nanostructures can also facilitate charge separation and transport processes within the solar cell by modifying the local electric field and providing additional pathways for charge carriers.

Given the significant progress made in the field, this review aims to provide a comprehensive overview of the latest research advancements in plasmonic nanostructures for enhancing the performance of PSCs. We will delve into the fundamental principles of surface plasmon polaritons (SPPs) and their excitation in various plasmonic nanostructures. Furthermore, we will examine the various strategies employed to integrate plasmonic nanostructures into PSC architectures, including nanoparticle embedding, nanohole arrays, and hybrid plasmonic–photonic designs. The optical and electrical enhancements achieved through these approaches, along with their underlying mechanisms, will be critically analyzed. Finally, we will discuss the remaining challenges and future directions for the continued advancement of plasmonic-enhanced PSCs.

## 2. Mechanisms of Plasmonic Nanostructures for Enhanced Optical and Electrical Properties

**Figure 2:**
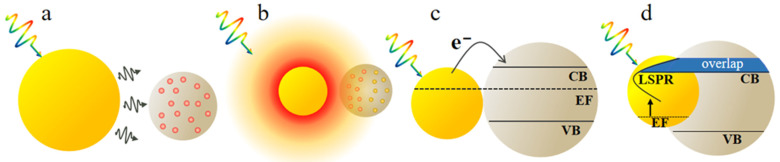
The four enhancement mechanisms of plasmonic nanostructures. (**a**) Far–field scattering. (**b**) Near–field coupling. (**c**) Hot–electron transfer. (**d**) Plasmon resonant energy transfer. Reproduced with permission from Ref. [72]. Copyright 2016, the Royal Society of Chemistry.

When the light wave is incident on the interface between metal and dielectric, the free electrons on the metal surface undergo collective oscillations, coupling with the electromagnetic wave to form a near-field electromagnetic wave propagating along the metal surface [71]. If the oscillation frequency of the electrons is consistent with the frequency of the incident light wave, resonance will occur, forming LSPR. At this time, the energy of the electromagnetic field is effectively converted into the collective oscillation energy of the free electrons on the metal surface, thereby causing unique optical phenomena and enhancing the near-field electromagnetic field on or near the metal surface. Common metal materials mainly include Au, Ag, Al, Cu, etc., because these metals have relatively superior plasmonic properties and storage abundance in nature. The enhancement of solar cell performance by plasmonic metal nanostructures is mainly attributed to two modes, radiation effect and non–radiation effect [72]. The radiation effect includes far-field scattering and near-field coupling effects, while the non-radiation effect includes hot–electron transfer (HET) and plasmon resonance energy transfer (PRET). As shown in Figure 2, the working principles of these four enhancement mechanisms are discussed.

### 2.1. Far–Field Scattering

The essence of far-field scattering lies in the scattering of light onto metal nanostructures with high reflectivity, entering the far field. Depending on the geometric properties of particles and material properties, the scattering cross–section may be an order of magnitude larger than the physical cross-section of the nanostructure. Even at distances of hundreds of nanometers, this far–field–scattered light can still be reabsorbed by the light absorption layer in solar cells [73]. Photon scattering from nanostructures in the far field undergoes multiple scattering from proximal nanostructures, ultimately enhancing the total amount of light captured in solar cells. The optical scattering properties of different types of MNPs are influenced by various factors, including size, shape, composition, and dielectric environment [74]. Therefore, we can adjust the LSPR to complement the wavelength range of the light absorption layer through carefully designed nanostructures, thereby achieving the reutilization of uncaptured photons and enhancing the light–harvesting efficiency of the device [75,76].

### 2.2. Near–Field Enhancement

When incident photons interact with plasmonic nanostructures and resonate, the electromagnetic field is confined within a small area on the metal surface and undergoes significant enhancement, known as near–field enhancement. In the direction perpendicular to the interface, the field strength of surface plasmons decays exponentially, but near the metal surface, its field strength is much higher than that of the incident light waves [77]. Surface plasmons have sub–wavelength localization, meaning their propagation is confined to a small region on the metal surface, leading to high energy concentration in the near-field region [78]. The enhancement effect mainly comes from the collective oscillation of free electrons on the metal surface, which binds and amplifies the electromagnetic waves. Strong near-field coupling effects occur around nanostructures, significantly enhancing the coupling between plasmas and molecules, thereby promoting the generation of electron-hole pairs in the active layer. Subsequently, these high-density charge carriers are effectively transferred to the TiO_2_, thereby increasing the overall current density of the solar cell. Similar to far-field scattering, the near field generated by LSPR is also influenced by the shape and composition of the metal nanostructures. In particular, non–spherical nanostructures with sharp features generate high charge concentrations at the edges and corners, leading to slower decay of the generated near field, extending it further.

### 2.3. Hot–Electron Transfer

The non–radiative dissipation of plasmon energy can generate hot electrons through Landau damping, which possess higher energies than those produced by thermal excitation. Compared to electrons directly generated through interband transitions in semiconductors, hot electrons transferred from excited plasmonic metals exhibit a higher thermodynamic driving force and lower charge recombination for photocatalytic redox reactions [79,80]. The hot electrons generated by plasmons can directly interact with molecules adsorbed on the metal surface. Furthermore, the hot electrons can also be transferred to adjacent semiconductor supports via plasmon-mediated electron transfer pathways. In plasmonic metal/semiconductor systems, the excitation of plasmons significantly enhances the yield of hot electrons with high potential energy on the plasmonic metal and induces the rapid and efficient transfer of these hot electrons to the semiconductor [81]. The Schottky barrier aids in capturing the transferred hot electrons in the conduction band of the semiconductor by delaying the return of the hot electrons to the plasmonic metal. This plasmon–mediated electron transfer strategy effectively prolongs the lifetime of the hot electrons transferred to the semiconductor conduction band, thereby enabling them to promote various surface chemical reactions. To maximize the effectiveness of hot electron injection, close contact between the metal and semiconductor is essential [82,83]. There are numerous factors influencing the generation and injection of hot electrons. Firstly, the size of the nanostructure plays a crucial role. Larger nanoparticles tend to produce hot carriers with very low excitation energies, whereas smaller metal particles often generate hot electrons that transition to higher energy levels. Additionally, smaller metal nanostructures favor the migration efficiency of hot electrons due to the limited migration speed of hot electrons in metals and their relatively short migration lifetime. It is estimated that the optimal size of gold nanoparticles ranges from 10 to 20 nm to maximize the generation and injection efficiency of hot electrons [84]. The second point concerns the shape of nanostructures. Theoretical calculations have shown that the hot electron injection rate of metallic nanorods is several orders of magnitude higher than that of spherical nanoparticles. With the aspect ratio remaining constant, the rate of hot electron injection into semiconductors increases as the aspect ratio of the nanorods increases but decreases as the volume of the nanorods increases. The third point pertains to the metal–semiconductor interface. Studies have indicated that the Schottky barrier height and defect states near the interface can impact the hot electron injection process. Strong near-field coupling at the plasmonic metal–semiconductor interface can facilitate hot electron transfer [85].

### 2.4. Plasmon Resonant Energy Transfer

PRET is a novel energy transfer mechanism that relies on dipole–dipole interactions between noble metal MNPs and energy acceptor molecules to facilitate energy exchange. When the optical field generated by the LSPR of noble MNPs matches the excited state energy level of a semiconductor at a certain distance, non–radiative energy transfer from the donor to the acceptor is achieved through dipole–dipole interactions [86]. This energy transfer process does not involve the emission or absorption of photons but is realized directly through electric or magnetic field interactions. Compared to radiative energy transfer methods such as fluorescence resonance energy transfer (FRET), the PRET process does not involve the emission or absorption of photons, thereby reducing energy loss and background interference. Furthermore, the directions of energy transfer are fundamentally different between the two: in PRET, energy is transferred from the plasmon to the semiconductor, whereas in FRET, energy is transferred from the semiconductor to the plasmon. For HET, a minimum separation of 2 nm is required between the metal and the semiconductor, whereas PRET is not affected by any insulating interlayer. As long as the semiconductor is within the near-field range of the metallic nanostructure, an interaction between the LSPR and the semiconductor absorption occurs, leading to spectral overlap. Moreover, the occurrence of HET necessitates the alignment of the metal’s Fermi level with the semiconductor’s energy bands, whereas PRET is not constrained by Fermi level equilibration. The plasma enhancement of PSCs by PRET depends on the morphology and composition of the metal nanostructures as well as the distance between the semiconductor and the metal nanostructures [87].

## 3. Types of Plasmonic Materials in PSCs

### 3.1. Noble Metal Plasmonic Nanomaterials

Noble metals Au and Ag are widely used to enhance the performance of PSCs due to their excellent plasmonic properties (resonance bands covering a broad range from visible to infrared regions of the solar spectrum). The local surface plasmon resonances of Au and Ag nanostructures are located near 520 nm and 400 nm, respectively, which can overlap with the absorption peaks of the active layer or ETL of solar cells, thereby promoting radiative and non-radiative coupling [77]. Although Au nanostructures exhibit visible band-to-band transitions and intraband transitions in the near-infrared (NIR) spectrum leading to high optical losses, their high stability, ease of synthesis, and direct visible light capture advantages still drive their application in PSCs [88,89]. Ag has low dielectric loss, determined by the imaginary part of the dielectric function [90,91], which results in Ag nanostructures having a higher extinction coefficient and greater light scattering [92,93], thereby increasing the light trapping efficiency of photovoltaic devices. The resonance bands of both are mainly located in the visible light region, and the stable nature of the material itself make it possible to customize nanostructures with shapes and sizes that meet the requirements through physical or chemical methods, thereby effectively improving the photovoltaic performance of PSCs. The following will be classified according to the different shapes of Au, Ag, and their core–shell nanoparticles and a detailed summary and analysis of the application research progress and action mechanism of different metal nanostructures in PSCs.

#### 3.1.1. Ag Nanomaterials

**Figure 3:**
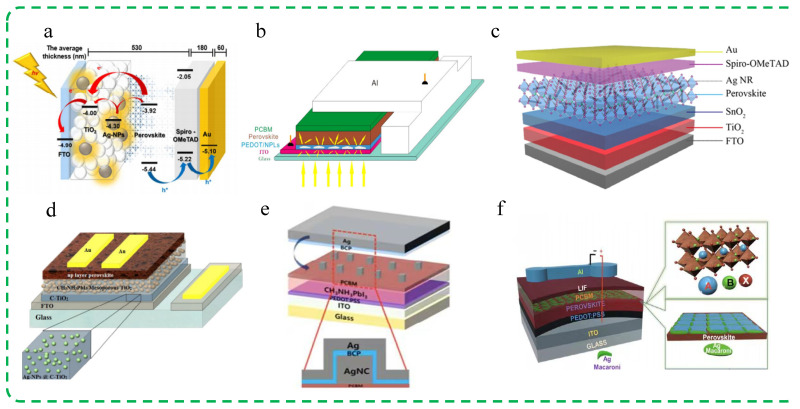
Summary of research for PSCs incorporating different Ag plasmonic nanostructures. (**a**) Ag nanospheres: Reproduced with permission from Ref. [94]. Copyright 2016, American Chemical Society. (**b**) Ag NPLs: Reproduced with permission from Ref. [95]. Copyright 2015, Elsevier. (**c**) Ag NRs: Reproduced with permission from Ref. [96]. Copyright 2020, Wiley. (**d**) Ag nanoparticles: Reproduced with permission from Ref. [97]. Copyright 2016, Elsevier. (**e**) Ag NCs: Reproduced with permission from Ref. [98]. Copyright 2017, American Chemical Society. (**f**) Ag NPLs: Reproduced with permission from Ref. [99]. Copyright 2020, Wiley.

Ag nanospheres. Yang et al. investigated the effect of neatly arranged spherical Ag nanospheres added to the TiO_2_ photoanode layer of PSCs on the device’s PCE [94]. The final results showed that a concentration of 0.5 wt% Ag nanospheres was optimal, which could achieve a PCE of 11.96% for the device. The intense plasmonic resonance response of Ag nanospheres enhanced a broad range of incident light absorption in the visible light wavelength region, improving charge transfer capability and beneficially enhancing the current density and photovoltaic conversion efficiency of PSCs. Meanwhile, the densely arranged Ag nanospheres on the TiO_2_ layer reduce its surface roughness and also contribute to the improvement of device performance. The schematic diagram, energy level diagram, and average thickness value of the device structure for PSCs are shown in Figure 3a.

Ag nanoplates (NPLs). Hsiang et al. adopted a wet chemical method to synthesize Ag NPLs with controllable shapes and sizes, with a thickness of 12 ± 4 nm and a side length of 70 ± 20 nm, supporting an SPR wavelength range of 500–750 nm [95]. With the increase in the edge length of Ag NPLs, the SPR band becomes broader, and the supported plasmonic effect also changes accordingly. By incorporating prepared Ag NPLs into the HTL of PSCs, the device’s PCE increased from the initial 8.5% to 9.6%, resulting in an overall improvement of 12.9%. This is attributed to the broad-band resonant absorption supported by the plasmonic Ag NPLs in the PSCs, leading to enhanced light harvesting of the photovoltaic device and thus improving efficiency. The schematic diagram of the PSCs device with Ag NPLs added is shown in Figure 3b.

Ag nanorods (NRs). Liu et al. introduced Ag NRs into the perovskite precursor solution by injecting the Ag NRs’ water solution, thus incorporating the Ag NRs into the perovskite active layer [96]. The Ag NRs increase the light absorption cross-section and scattering cross-section through LSPR, ultimately increasing the probability of the perovskite layer capturing light, thereby enhancing the short-circuit current density (*J*_SC_) of the device. At the same time, the presence of appropriate water molecules helps to obtain high-quality larger grain size and less defective perovskite films, significantly increasing the fill factor (FF) of the device. The results show that Ag NRs have a good synergistic effect with water molecules, successfully reducing the defect density of the perovskite layer and significantly improving the mobility. When the added concentration reaches 2 vol%, the device performs best, with *J*_SC_ at 22.18 mA/cm^2^, FF at 81.68%, and PCE at 20.29%. Compared to the standard device without the addition of Ag NR solution, the performance parameters of the photovoltaic device have increased by 5.2%, 4.2%, and 9.7%, respectively. The device structure schematic diagram with the addition of Ag nanorod aqueous solution is shown in Figure 3c.

Ag nanoparticles. Precious MNPs can effectively enhance the light trapping efficiency of photovoltaic devices through the excitation of LSPR under illumination. Nourolahi et al. embedded plasmonic Ag nanoparticles into the TiO_2_ layer of mesoporous heterojunction PSCs to study their impact on the device’s photovoltaic characteristics [97]. The final results showed that compared to samples without added Ag nanoparticles, the PCE of the fabricated cells increased by over 30%. According to the analysis of UV–visible absorbance data, the improved performance can be attributed to the far-field scattering and near-field coupling radiation effects of the Ag nanoparticles doped in the visible wavelength range of the solar spectrum, effectively extending the optical path of the incident light and increasing the photon absorption of the active perovskite layer. At the same time, the electron transfer time between the TiO_2_ interface layer and the photovoltaic perovskite layer is reduced due to the improved frequency response of the electron transfer behavior in the photoelectric anode interface layer. The schematic diagram of the structure of the PSCs with added Ag nanoparticles is shown in Figure 3d.

Ag nanocubes (NCs). By enhancing the light absorption of the edge region of nanoparticles in plasmas, the PCE of high-efficiency solar cells can be further improved. Kim et al. have developed a planar PSCs with plasmonic Ag NCs electromagnetically coupled to a Ag back electrode, achieving the resonance wavelength control of the electrode-coupled plasmas [98]. By adjusting the thickness of the electron transfer layer inserted between the NCs and the electrode, the plasmon peak wavelength can be adjusted to the absorption edge of the perovskite active layer (600–800 nm). Due to the coupling with the Ag back electrode, the plasmonic resonance of Ag NCs is enhanced, leading to a significant improvement in far-field scattering and optical near-field originating from the nanocube faces closest to the perovskite layer. The electrode-coupled plasmon enhances the photocurrent at the edge of the PSCs, resulting in an increase in the average PCE from 11.9% to 13.3%. The schematic diagram of the PSC structure with electrode-coupled Ag NCs is shown in Figure 3e.

Ag NPLs. Ali et al. studied the effect of adding Ag NPLs based on vapor deposition to the perovskite active layer on device performance [99]. The final results show that the PCE of the device with Ag NPLs of size 79 ± 6 nm added increased from 11.63% to 13.46%, with an improvement factor of 15.74%. In addition, the *J*_SC_ of the device also increased. Based on the LSPR effect, Ag NPLs exhibit the ability to enhance both near-field and far-field, scattering enhanced absorption and increasing the optical path length of photons in the active layer, effectively improving the photon–electron conversion efficiency of PSCs. In addition, the UV photoelectron spectroscopy shows a reduction in the hole injection barrier, which also helps to improve the device performance. As shown in Figure 3f, a schematic diagram of a photovoltaic device structure containing Ag NPLs.

**Figure 4:**
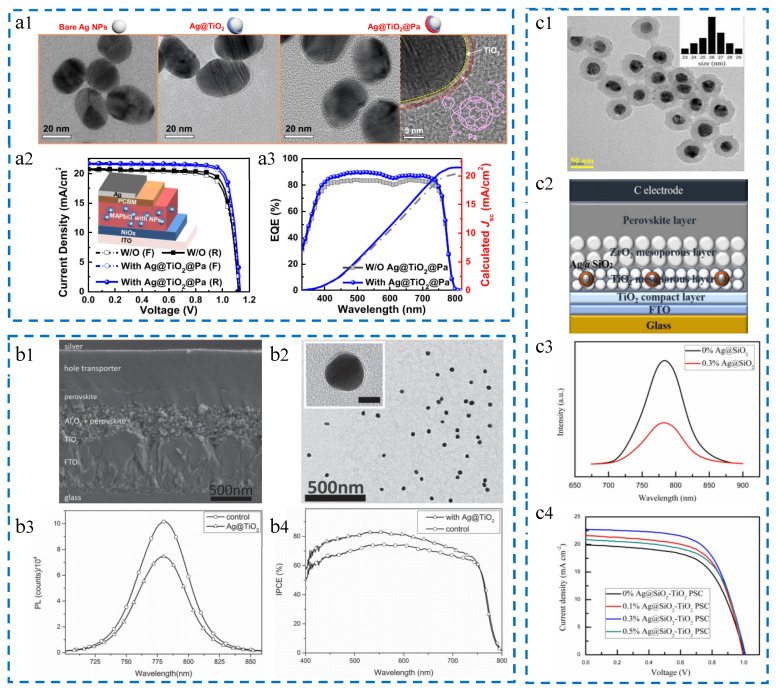
Summary of research for PSCs incorporating different Ag core–shell plasmonic nanostructures. (**a1**–**a3**) Ag@TiO_2_@Pa nanoparticles: Reproduced with permission from Ref. [100]. Copyright 2019, American Chemical Society. (**b1**–**b4**) Ag@TiO_2_ nanoparticles: Reproduced with permission from Ref. [101]. Copyright 2015, Wiley. (**c1**–**c4**) Ag@SiO_2_ nanoparticles: Reproduced with permission from Ref. [102]. Copyright 2018, MDPI.

Ag@TiO_2_@Pa nanoparticles. Yao et al. added Ag@TiO_2_@Pa nanoparticles to the active layer of PSCs, improving light absorption and charge carrier extraction, ultimately resulting in increased *J*_SC_ and FF [100]. As shown in Figure 4(a1), a TEM image of the prepared nanoparticles, the size of the Ag@TiO_2_@Pa nanoparticles is 28 nm. With the synergistic effect of light effect and electrical effect, the addition of nanoparticles increases the efficiency of PSCs from 18.4% to 20.2%, an increase of 10.2%. As shown in Figure 4(a2,a3), the current density–voltage (*J–V*) curves and EQE spectra of the optimized reference device and plasma device are provided. The improvement in the efficiency of the photovoltaic device is attributed to the addition of core–shell nanoparticles, which reduce the losses when bare Ag nanoparticles are dispersed in the active layer; and it is also attributed to the field effect of LSPR and effective light scattering, increasing the generation of excitons and improving light absorption.

Ag@TiO_2_ nanoparticles. Michael et al. successfully incorporated core–shell Ag@TiO_2_ nanoparticles into PSCs through a low-temperature processing approach, leading to an increase in device efficiency to 16.3% [101]. This enhancement is mainly attributed to the increase in *J*_SC_. As shown in Figure 4(b1,b2), the scanning electron microscope (SEM) cross-section of the solar cell and the transmission electron microscope (TEM) image of Ag@TiO_2_ nanoparticles, where the Ag@TiO_2_ nanoparticles are composed of a 40 nm Ag core and a 2 nm TiO_2_ shell, can be observed. Figure 4(b3,b4) separately represents the steady-state photoluminescence spectrum and the photovoltaic conversion efficiency of the reference device and the device with added Ag@TiO_2_ nanoparticles. It can be seen from the figure that the addition of nanoparticles does improve the performance of the photovoltaic device, especially in the visible light range of 400–750 nm.

Ag@SiO_2_ nanoparticles. Wang et al. synthesized Ag@SiO_2_ nanoparticles using an improved oxidation method and integrated them into the mesoporous TiO_2_ layer of PSCs [102]. This ultimately led to an increase in the PCE of the cell from 12.23% to 14.61% and an increase in *J*_SC_ from 20.23 mA/cm^2^ to 23.04 mA/cm^2^. These enhancements are mainly attributed to the LSPR effect and strong scattering effect of Ag@SiO_2_ nanoparticles. As shown in Figure 4(c1,c2), the TEM image and schematic structure of solar cells with Ag@SiO_2_ nanoparticles are presented, where the size of the nanoparticles is 40 nm. Figure 4(c3) shows the photoluminescence spectrum of the device at room temperature, indicating that the addition of nanoparticles promotes the exciton ionization and charge separation processes. Figure 4(c4) displays the *J–V* curves of the cells with different contents of Ag@SiO_2_ nanoparticles added. It can be observed from the graph that the addition of nanoparticles is beneficial for improving the device performance, with the best effect achieved at a content of 0.3%.

#### 3.1.2. Au Nanomaterials

**Figure 5:**
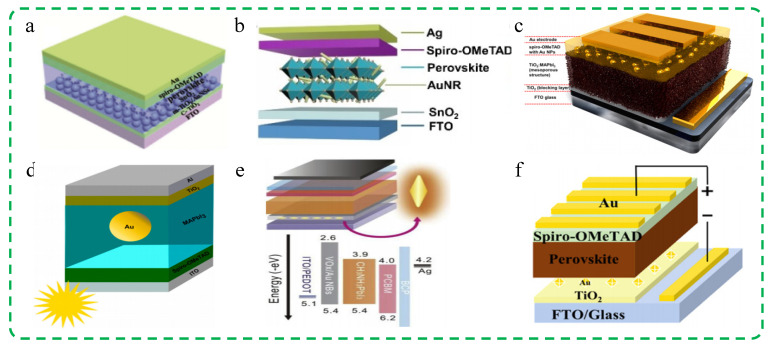
Summary of research for PSCs incorporating different Au plasmonic nanostructures. (**a**) Au NSs: Reproduced with permission from Ref. [103]. Copyright 2019, Elsevier. (**b**) Au NRs: Reproduced with permission from Ref. [104]. Copyright 2021, Wiley. (**c**) Au nanoparticles: Reproduced with permission from Ref. [105]. Copyright 2015, American Chemical Society. (**d**) Au nanosphere: Reproduced with permission from Ref. [106]. Copyright 2019, MDPI. (**e**) Au NBs: Reproduced with permission from Ref. [107]. Copyright 2018, Elsevier. (**f**) Au NOs: Reproduced with permission from Ref. [108]. Copyright 2021, American Chemical Society.

Au nanostars (Au NSs). Wu et al. incorporated 40 nm Au NSs into the ETL of ultra-thin PSCs, enhancing light harvesting and charge carrier transport [103]. The schematic structure of the cell device is shown in Figure 5a. To prevent direct contact between Au NSs and the perovskite active layer, a TiO_2_/SnO_2_ dual electron–modifying layer was introduced. In addition, the impact of Au NSs on the PCE of PSCs with different thicknesses was systematically studied, revealing varying improvements in PCE for different thicknesses of the perovskite layer with the introduction of the same Au NSs. For ultra–thin PSCs with a 250 nm perovskite layer, the PCE increased from 15.34% to 18.50%, with an enhancement factor of 20.60%; while for PSCs with a 400 nm perovskite layer, the PCE increased to 20.06%, with an enhancement factor of 13.70%.

Au nanorods (NRs). Gao et al. used the Asynchronous Synergistic Effect (ASE) strategy of water and Au NRs, adding a solution of Au NRs in water to the precursor solution of perovskite [104]. As shown in Figure 5b is the schematic structure of the cell device. The former improved the quality of the perovskite film during the crystallization process, reducing the defect density and increasing the carrier mobility. At the same time, when the device is exposed to light, the latter increases the light absorption of the perovskite layer through the LSPR effect. The results show that the ASE strategy has an excellent PCE of 21.73% and excellent long–term stability. After storing in air for 3 months, it can maintain 95% of the initial PCE.

Au nanoparticles. Lee et al. embedded Au nanoparticles into the HTL of PSCs to study the photovoltaic effect between PSC and Au nanoparticles [105]. Figure 5c shows the schematic structure of the cell device. The results show that Au nanoparticles cause near-field coupling in the short wavelength range, increasing *J*_SC_. This is attributed to the LSPR effect and electron effect exhibited by Au nanoparticles. By comparing backscattered electron (BSE) data and atomic force microscope (AFM) images, it is shown that Au nanoparticles are located near the perovskite layer inside the HTL of PSCs. This implies that the improvement of EQE in PSCs by Au nanoparticles is a result of optical plasmonic effects and electronic contributions in a wide wavelength range.

Au nanosphere. Elnaz and colleagues investigated the influence of adding spherical Au nanoparticles with different radii (20–60 nm) into the active layer of PSCs (MAPbI_3_) on their light absorption [106]. As shown in Figure 5d is the schematic structure of the cell device. It was found that the addition of 60 nm radius Au nanoparticles increased the light absorption of the cell by 20%. This is because placing plasmonic nanospheres in the device enhances the electric field around the nanoparticles, increasing the possibility of light absorption within the active layer. At the same time, both the amplitude and width of the absorbance spectrum of the PSCs were improved. As the radius of the nanoparticles increases, scattering is enhanced, leading to an overall increase in the cell’s absorption.

Au nanobipyramids (NBs). Dong et al. prepared Au NB structure and incorporated it into the HTL of planar heterojunction PSCs, the schematic structure of the cell device is shown in Figure 5e [107]. This typical metallic nanostructure exhibits multiple and strong plasmonic absorption characteristics from visible light to NIR bands, showing a higher plasmon-induced probability. In addition, compared to traditional Au nanoparticles, the Au NBs show stronger electromagnetic field enhancement, thereby enhancing both light harvesting and improved interface charge dynamics. The final results show that the PCE of the best photovoltaic device with Au NBs is 18.84%, while the reference device’s PCE is only 16.02%. At the same time, Au NBs also improve the electrical performance of the device. Under operating conditions, Au NBs effectively fill the hot hole injection, helping to increase the open circuit voltage, eliminate hysteresis effects, and improve long–term stability.

Au nano-octahedrons (NOs). Fang et al. synthesized Au NOs with broadband LSPR peaks and appropriate sizes and added them to the ETL (TiO_2_) of PSCs [108]. Figure 5f shows the schematic structure of the cell device. The results showed that the *J*_SC_ of the cell reached 23.63 mA/cm^2^, and the PCE increased from 16.95% to 19.05%. This is because the LSPR of the nanostructure enhanced the light capture of the cell, improving the device’s light harvesting capability and carrier extraction. Au NOs can enhance the light absorption of perovskite layers by increasing the light-scattering cross-section, and perovskite layers can effectively excite more excitons. In addition, Au NOs also lower the surface potential of ETL, facilitating the extraction and transfer of electrons and holes photo-generated at the ETL/perovskite layer interface. Therefore, both *J*_SC_ and *V*_OC_ of PSCs have been significantly improved, leading to a corresponding enhancement in PSCs’ efficiency.

**Figure 6:**
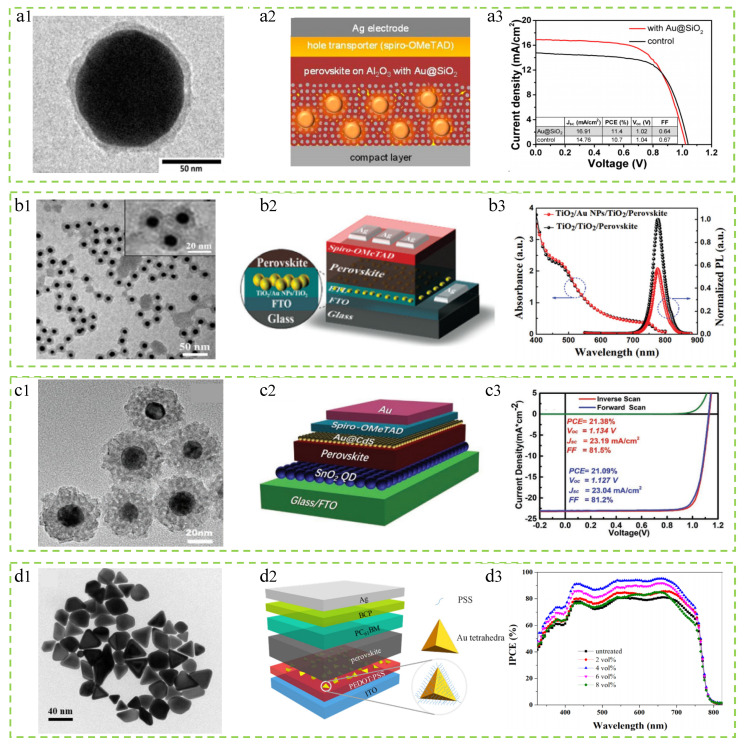
Summary of research for PSCs incorporating different Au core–shell plasmonic nanostructures. (**a1**–**a3**) Au@SiO_2_ nanoparticles: Reproduced with permission from Ref. [109]. Copyright 2013, American Chemical Society. (**b1**–**b3**) Au@SiO_2_ nanoparticles: Reproduced with permission from Ref. [110]. Copyright 2020, the Royal Society of Chemistry. (**c1**–**c3**) Au@CdS nanoparticles: Reproduced with permission from Ref. [111]. Copyright 2020, Wiley. (**d1**–**d3**) Au@PSS nanoparticles: Reproduced with permission from Ref. [112]. Copyright 2018, Wiley.

Au@SiO_2_ nanoparticles. Zhang et al. prepared Au@SiO_2_ core–shell nanoparticles and integrated them into the ETL of PSCs [109]. The TEM image of the nanoparticles and the schematic structure of the cell device are shown in Figure 6(a1,a2). The final results show that the addition of Au@SiO_2_ nanoparticles enhances the photocurrent and efficiency of the solar cells, reaching a device efficiency of 11.4%. The optimal performance *J–V* curve of the battery is shown in Figure 6(a3). In contrast to other studies, the improved *J*_SC_ is attributed to the decrease in exciton binding energy, that is, the addition of MNPs enhances the generation of free charge carriers rather than enhancing light absorption.

Au@SiO_2_ nanoparticles. The Au@SiO_2_ nanoparticles prepared by Cui et al. are dispersed between two consecutive TiO_2_ ETLs of PSCs [110]. This will result in the process of HET, where the hot electrons of the Au core can easily be injected into TiO_2_, thereby promoting the local electron migration of the TiO_2_, leading to improved charge transport and increased *J*_SC_. At the same time, the HET effect rises to the Fermi level of TiO_2_, resulting in enhanced built-in potential and open-circuit voltage (*V*_OC_). Finally, efficiencies of 18.81% and 19.42% were achieved in planar and mesoscopic structure PSCs, respectively. Figure 6(b1) shows the TEM image of Au@SiO_2_ core–shell nanoparticles, revealing a nanoparticle size of 18 nm. Figure 6(b2) depicts the structure of the PSCs. Figure 6(b3) UV–visible absorption and time-integrated PL spectra indicate a significant decrease in PL due to the addition of nanoparticles, suggesting charge transfer from perovskite to TiO_2_.

Au@CdS nanoparticles. Qin et al. synthesized core–shell Au@CdS nanospheres, dispersed between the active layer and the HTL of PSCs, with a nanoparticle size of 35 nm [111]. Figure 6(c1,c2) show the high-resolution transmission electron microscope (HR-TEM) image of the nanospheres and the structural diagram of the PSC device, respectively. With the LSPR effect of Au@CdS, holes can easily overcome barriers at the perovskite interface, avoid carrier accumulation, suppress carrier trap recombination at the Spiro-OMeTAD/perovskite interface, effectively improve the generation/dissociation of excitons, and promote the transfer/collection of carriers. Therefore, the PSC device based on Au@CdS achieved an efficiency of over 21%.

Au@PSS nanoparticles. Hao et al. incorporated Au@PSS nanoparticles prepared into PSCs to enhance the light absorption of lead iodide (PbI_2_) through the surface plasmon resonance effect [112]. The use of tetrahedral Au cores and the introduction of ultra-thin PSS shell layers are both beneficial for generating a strong localized field and preventing exciton quenching on the surface of the nanoparticles. At the optimal concentration of Au@PSS nanoparticles, the PCE reached 16.53%, with a significant enhancement factor of 18.83% compared to the reference device without nanoparticles. The results indicate that in addition to promoting light absorption in the active layer, Au@PSS nanoparticles also enhance the composite resistance inside PSCs and improve exciton dissociation and charge transfer efficiency by reducing the intensity of photoluminescence and the lifetime of excitons/carriers.

In addition to the noble metal geometrical shapes discussed in the above figures, we also provide a comprehensive list in Table 1, including a range of shape-controlled noble metal and their core–shell nanostructures that have recently been used in PSCs in the literature. This provides a reference for further exploring the working mechanism of noble metal plasmonic nanostructures, enhancing PSCs, and further perfecting the theoretical system of the application of LSPR effects in PSCs.

### 3.2. Other Metal Plasmonic Nanomaterials

#### 3.2.1. Al Nanomaterials

In addition to precious metals such as Au and Ag, Al is recommended as a new alternative plasma material due to its advantages of low cost, abundant natural reserves, and broad response range in plasma spectroscopy [122,123]. It has a plasma frequency of up to about 15.3 eV, much higher than Ag’s 9.6 eV and Au’s 8.55 eV [124], so it can extend the surface plasma response to the ultraviolet region and support long-lived LSPR with high optical cross-section. Meanwhile, unlike Au and Ag, the work function of Al (4.06 eV) is lower than that of TiO_2_ (4.26 eV), so using Al significantly reduces the expected quenching through charge transfer and carrier recombination, effectively improving the extraction efficiency of charge carriers. Additionally, due to low light loss and minimal plasmon damping, Al nanoparticles have higher light-scattering efficiency in visible light compared to Au and Ag [125]. In addition, a thin layer of natural oxide formed on the surface of Al can prevent further oxidation and contamination inside, thus ensuring the stability of the material itself. At the same time, it can avoid direct contact between the Al metal itself and the active layer and ETL of solar cells, reducing the generation of harmful radiation. Arul et al. increased the PCE of the device by 14% by placing Al nanoparticles with particle sizes ranging from 20 to 70 nm between the ETL and the electrode [126]. The addition of Al nanoparticles exhibited a dual effect, namely, local surface plasmon resonance and far-field scattering, thereby improving photon absorption in the active layer and increasing the photovoltaic conversion efficiency of PSCs. At the same time, the electrical conductivity and *J*_SC_ of the device have also been improved. It is also observed that the presence of Al nanoparticles in the ETL slightly reduces the deep trap density, while there is no change in the shallow trap state, thereby increasing the carrier concentration of the device.

#### 3.2.2. Cu Nanomaterials

Similar to the advantages of Al materials, Cu is also one of the materials that receive considerable attention besides noble metals such as Au and Ag. On the one hand, this is because of its low material cost, and, on the other hand, it displays good plasma characteristics in the visible light wavelength region, possessing photovoltaic properties similar to Au and Ag. If Cu nanoparticles can be successfully used to enhance the efficiency of PSCs, it can reduce manufacturing costs while maintaining the light absorption of solar cells, thereby promoting the development of solar cells and achieving commercial production sooner. Shreya et al. simulated the effect of adding Cu nanoparticles to PSCs using the Finite Difference Time Domain (FDTD) method and conducted a rigorous analysis of the size and position of the nanoparticles [127]. It was ultimately found that adding spherical nanoparticles with a radius of 70 nm at the center of the 200 nm perovskite layer can achieve the maximum enhancement effect.

### 3.3. Alloy Plasmonic Nanomaterials

Apart from size and morphology, the composition also affects the LSPR properties of plasma nanoparticles. In order to prepare nanoparticles that meet the requirements, researchers have designed composite structures of dual materials, with the most typical being Au-Ag alloy nanostructures. It is well known that Au and Ag nanoparticles themselves have strong plasmonic properties, so the coupling effect of the two materials will lead to a further enhancement of the plasmon resonance mode [128,129], characterized by an increase in the number of LSPR extinction peaks. Compared to single MNPs, alloy nanoparticles have greatly improved absorption and scattering intensities and broader and stronger plasmon resonance effects, ultimately achieving enhanced broadband absorption [130]. In addition, due to the strong coupling between the two metals, the intensity of the near-field electromagnetic field of alloy nanoparticle LSPR is significantly increased [131], which can reduce the number of MNPs used in plasmonic solar cells, thereby improving material utilization and reducing production costs. Therefore, alloy nanoparticles are of great significance for enhancing the performance of plasmonic PSCs.

**Figure 7:**
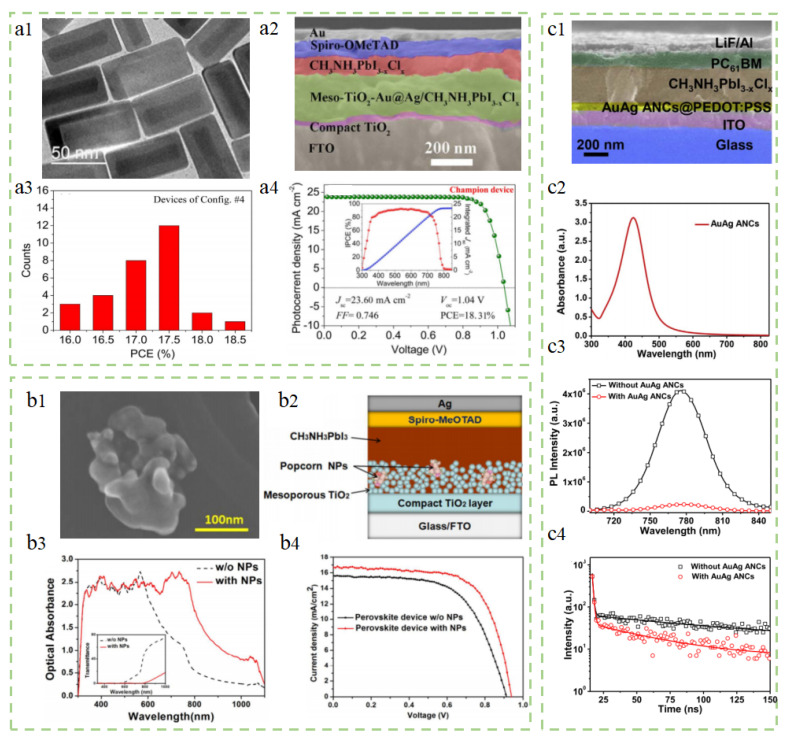
Summary of research for PSCs incorporating different alloy nanomaterials. (**a1**–**a4**) Au@Ag nanocuboids: Reproduced with permission from Ref. [132]. Copyright 2017, Elsevier. (**b1**–**b4**) Au–Ag alloy popcorn-shaped nanoparticles: Reproduced with permission from Ref. [133]. Copyright 2012, the Royal Society of Chemistry. (**c1**–**c4**) AuAg alloy nanocrystals: Reproduced with permission from Ref. [134]. Copyright 2017, Elsevier.

Au@Ag nanocuboids. Fu et al. synthesized Au@Ag-alloy-encapsulated nanocuboids. Compared to traditional metal nanostructures with a single narrow plasmon resonance band, Au@Ag nanoparticles exhibit multiple broader and stronger plasmon resonances [132]. By adjusting the structural size to match the spectral absorption band of perovskite, especially in its weak absorption region. By adjusting the position of Au@Ag nanoparticles in the electrode, the performance of PSCs can be effectively improved by utilizing the plasmonic near-field enhancement and increased light-scattering effects. Figure 7(a1,a2) show the TEM images of Au@Ag core–shell nanocuboids and the cross-sectional SEM image of PSCs with added Au@Ag core–shell nanocuboids, respectively. Figure 7(a3,a4) represent the statistically averaged PCE of the devices and the parameter curves of the best-performing device. It can be observed that the device’s PCE is concentrated around 17.5%, while the champion PCE of the best plasmonic PSCs is 18.31%.

Au-Ag alloy popcorn-shaped nanoparticles. Lu et al. prepared Au-Ag alloy popcorn-shaped nanoparticles and placed them in the ETL of PSCs [133]. The TEM image of the popcorn-shaped nanoparticles is shown in Figure 7(b1), with an average size of 150 ± 50 nm. The unique popcorn nanoparticles have the advantage of exciting various LSPR modes in a broader range of solar spectral. As shown in Figure 7(b3,b4), the optical absorption curve with added nanostructures and the *J–V* curve of the best-performing perovskite device are presented. It can be observed from the figures that the addition of popcorn-shaped nanoparticles not only broadens the device’s absorption spectrum of solar light but also effectively improves the *J*_SC_ of the device. Therefore, by using these nanoparticles, the light absorption of PSCs can be significantly enhanced, and the photoinduced charge transfer can be improved. As a result, the maximum PCE of the device increased from 8.9% to 10.3%, representing a 15.7% increase.

AuAg alloy nanocrystals. Sun et al. synthesized AuAg core–shell alloy nanocrystals (ANCs) with a size of 46 nm, doped into the HTL (PEDOT: PSS) of PSCs [134]. Figure 7(c2) shows a cross-sectional SEM image of the device with added nanoparticles. Figure 7(c1) shows the UV–visible absorption spectrum of AuAg ANCs, displaying a dense characteristic absorption peak at 428 nm. Figure 7(c3,c4) show steady-state and time-resolved PL spectra of perovskite films with and without AuAg alloy nanoparticles on the PEDOT: PSS layer. The image shows that the perovskite film with added nanoparticles exhibits a significant PL quenching, indicating enhanced charge transfer from the perovskite film to the HTL. Ultimately, the device doped with nanoparticles achieved a champion PCE of 16.76%, a 28% increase compared to the undoped device (13.14%). The study shows that the significant improvement in device performance is primarily attributed to the scattering effect of AuAg alloy nanocrystals that improve light collection and optimize charge transfer in the HTL.

### 3.4. Other Novel Plasmonic Nanomaterials

**Figure 8:**
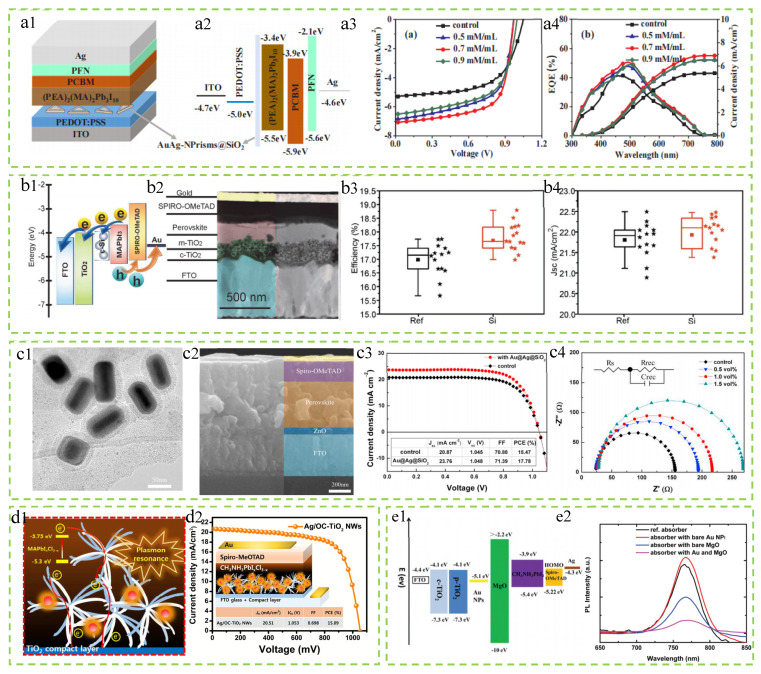
Summary of research for PSCs incorporating novel plasmonic nanomaterials. (**a1**–**a4**) AuAgNPrisms@SiO_2_: Reproduced with permission from Ref. [135]. Copyright 2019, Elsevier. (**b1**–**b4**) Si nanoparticles: Reproduced with permission from Ref. [136]. Copyright 2018, Wiley. (**c1**–**c4**) Au@Ag@SiO_2_ core–shell nanocuboids: Reproduced with permission from Ref. [137]. Copyright 2019, Elsevier. (**d1**,**d2**) SiO_2_@Ag@OC–TiO_2_ nanowires: Reproduced with permission from Ref. [138]. Copyright 2016, the Royal Society of Chemistry. (**e1**,**e2**) Au nanoparticles and MgO: Reproduced with permission from Ref. [139]. Copyright 2017, the Royal Society of Chemistry.

AuAgNPrisms@SiO_2_. Han et al. successfully prepared SiO_2_-coated AuAg-alloyed nanoprisms and evenly distributed them between the HTL and active layer of inverted PSCs [135]. The final results show that the best-performing device has *J*_SC_ and PCE of 7.08 mA/cm^2^ and 4.60%, respectively, which are 32.8% and 34.1% higher than the control group (*J*_SC_ = 5.33 mA/cm^2^ and 3.43%). As shown in Figure 8(a3,a4), the *J–V* curves and external quantum efficiency (EQE) curves with different concentrations of AuAgNPrisms@SiO_2_ nanoparticles are presented. It can be observed from the figures that the addition of nanoparticles significantly increases *J*_SC_ while slightly decreasing the *V*_OC_. Among the devices with three doping concentrations, the device with a concentration of 0.7 mM/mL performs the best. Meanwhile, the addition of AuAgNPrisms@SiO_2_ nanoparticles reduces the series resistance (Rs), leading to a significant increase in FF. The enhancements in *J*_SC_ and FF outweigh the decrease in *V*_OC_, resulting in an increased PCE of PSCs. In addition, the added AuAgNPrisms@SiO_2_ nanoparticles can act as inducers to enhance the smoothness of the perovskite film, increase light absorption, promote exciton dissociation, and facilitate charge carrier transport.

Si nanoparticles. Aleksandra et al. studied the effect of adding resonant Si nanoparticles between the TiO_2_ transport layer and the active layer on the performance of PSCs [136]. Figure 8(b1,b2) show the band diagram and cross–sectional bright–field STEM image of the PSCs, where the size of the added silicon nanoparticles is 140 nm. Figure 8(b3,b4) show the efficiency and *J*_SC_ data of the photovoltaic devices. According to the data in the figure, it can be seen that the PCE of all devices is above 15%, with the highest PCE of the optimal device being 18.8%. Compared with batteries without added nanoparticles, the presence of Si nanoparticles significantly improves the average *J*_SC_ and optimal *J*_SC_ of the batteries. The final research results show that the photocurrent and FF of PSCs have also been enhanced, reaching 22.4 mA/cm^2^ and 78.9% respectively. This is attributed to the plasmonic effect exhibited by Si nanoparticles, which leads to near-field enhancement, ultimately increasing the light absorption of the active layer and improving the photovoltaic performance of the solar cell.

Au@Ag@SiO_2_ core–shell nanocuboids (Au@Ag@SiO_2_ NCs). Deng et al. successfully embedded Au@Ag@SiO_2_ core–shell nanoparticles into the perovskite film of planar heterojunction PSCs, which significantly increased the *J*_SC_ [137]. The *J*_SC_ of the original device is 20.87 mA/cm^2^, while the *J*_SC_ with the addition of the Au@Ag@SiO_2_ NC device increased to 23.76 mA/cm^2^, an increase of 13.85%. This is because the addition of nanoparticles enhances the induced light absorption and charge carrier separation capabilities, improving the performance of the device. Figure 8(c1,c2) show the TEM image of Au@Ag@SiO_2_ NCs and SEM images of the cross-section of PSCs, with the size of the nanoparticles being 50 nm. Figure 8(c3) shows the *J–V* curves of the reference device and the device with added Au@Ag@SiO_2_ NCs. It can be seen that the addition of nanocuboids effectively improved the performance of the photovoltaic device, with the best–performing device having *J*_SC_ of 23.76 mA/cm^2^, *V*_OC_ of 1.048 V, FF of 71.39%, and PCE of 17.78%. Figure 8(c4) presents the Nyquist plots and simplified equivalent circuits of devices with different additional Au@Ag@SiO_2_ nanoparticle content. The results show that the addition of nanoparticles reduces the carrier recombination rate and promotes charge separation and carrier transport, leading to an increase in *V*_OC_.

SiO_2_@Ag@OC-TiO_2_ nanowires (SiO_2_@Ag@OC-TiO_2_ NWs). Yu et al. synthesized Ag@OC-TiO_2_NWs (SiO_2_@Ag@OC-TiO_2_ NWs) composite material encapsulated with Si and incorporated it into the ETL of PSCs [138]. As shown in Figure 8(d1), the figure illustrates the electron transfer path in the PSCs with Ag@OC-OTiO_2_NWs incorporated, as well as the plasmonic effect of Ag NPs decorated on OC-TiO_2_NWs. The LSPR and induced exciton dissociation mechanisms exhibited by Ag nanoparticles can significantly enhance the PCE of PSCs. Figure 8(d2) shows the optimal *J–V* curve of the PSCs after the incorporation of SiO_2_@Ag@OC-TiO_2_NWs, with the inset providing a schematic illustration of the structure of PSCs containing SiO_2_@Ag@OC-TiO_2_NWs. Due to the large pore structure in the OC-TiO_2_NWs, the permeability of the perovskite solution is enhanced, minimizing harmful voids in the ETL, which in turn achieves a higher *V*_OC_ and FF. Simultaneously, the plasma effect significantly enhances light absorption capabilities, and the increase in recombination resistance synergistically promotes the enhancement of current density. Ultimately, our research reveals that the PSCs based on the synergistic scaffold structure composed of Ag nanoparticles and OC-TiO_2_NWs exhibit exceptionally superior performance, achieving a PCE of up to 15.09%, marking a remarkable 24% enhancement compared to PSCs based on a scaffold layer of pure TiO_2_ nanoparticles. Furthermore, the average PCE of these PSCs with the synergistic scaffold structure also reaches 12.17%, demonstrating good stability and consistency.

Au nanoparticles and MgO. Zhang et al. successfully fabricated solar cells by incorporating Au nanoparticles into mesoporous TiO_2_ films and depositing a MgO passivation layer on the Au NP-modified mesoporous titanium substrate through a wet spinning process and the thermal decomposition of magnesium salt [139]. This approach aimed to enhance the performance of solar cells by improving interfacial properties and light absorption efficiency. The PSCs prepared by combining Au nanoparticles with MgO exhibit outstanding performance, with a PCE of up to 16.1%, a *V*_OC_ of up to 1.09 V, and a *J*_SC_ reaching 21.76 mA/cm^2^. Figure 8(e1) depicts the energy diagram of the materials in the PSCs containing Au nanoparticles and MgO. Figure 8(e2) shows the PL spectrum of the perovskite absorber layer with p-TiO_2_ or MgO coating. It can be clearly seen from the data in the figure that the PL quenching degree of perovskite samples with magnesium salt as MgO coating is significantly enhanced. This phenomenon confirms that the electron extraction efficiency of perovskite modified by MgO on the TiO_2_ layer is significantly higher than that of the original TiO_2_. Furthermore, when perovskite samples are simultaneously modified by Au nanoparticles and MgO, their PL peak intensity is reduced to the lowest, showing the most intense PL quenching effect. This indicates that the charge transfer process is significantly accelerated, effectively improving the electron extraction efficiency, reducing charge accumulation, and thus helping to increase the *J*_SC_ and overall photovoltaic performance of the device. Compared with devices based on pure TiO_2_, the PCE of the device has significantly increased by 34.2%. This enhancement may be closely related to the effective photon management generated by Au nanoparticles and MgO layers. This mechanism helps to reduce the photon and energy losses of charge carriers during the generation process, thereby enhancing the high charge transfer capability of PSCs and reducing charge recombination losses. In addition, by combining the use of Au NPs and insulating MgO layers in the passivation layer, the stability of PSC devices under ultraviolet light irradiation is significantly improved.

As shown in Table 2, a summary of the application of some plasma structures other than precious metals such as Au and Ag in PSCs is presented. This includes common metal Al, semiconductor material Si, and metal alloy nanomaterials Au-Ag and Cu-Ag, as well as some newly synthesized novel plasma structures such as AgOx@Ag nanoparticles, Au@PAT nanoparticles, etc.

## 4. Conclusions and Outlook

This review provides a comprehensive summary of recent advancements in the enhancement of PSCs through the incorporation of plasmonic nanostructures. Initially, the analysis delves into four prominent manifestations of LSPR employed in photovoltaic devices: far-field scattering, near-field enhancement, HET, and PRET. Subsequently, the applications of MNPs—comprising precious metals such as Au and Ag and common metals like Al and Cu, as well as various alloys—are systematically reviewed and analyzed based on their types and shapes. The results demonstrate that incorporating MNPs with different LSPR properties into PSCs can effectively improve the efficiency and overall performance of these cells. However, despite the significant advancements made thus far, several issues and challenges remain to be addressed for the widespread commercialization of plasmonic-enhanced PSCs:

(1) The selection of plasmonic materials that are both cost-effective and compatible with perovskite chemistry is crucial. At present, the primary materials employed are noble metals such as Au and Ag, which are both expensive and limited in supply. Consequently, there emerges an urgent imperative to scout and cultivate alternative materials that not only promise cost savings but are also abundant in nature. Al and Cu, albeit harboring potential as promising plasmonic materials, have been relatively neglected in the realm of research and application. Apart from these materials, we can develop a range of alternative plasmonic materials, such as transition metal materials, oxide materials, semiconductor materials, metal alloy materials, composite materials, and so on, to facilitate the further advancement of research on plasmonic solar cells. Moreover, the fabrication of plasmonic nanostructures encounters formidable challenges, encapsulated in substantial equipment expenditures, intricate manufacturing processes, substantial energy depletion, and the elusive pursuit of uniformity and stability. Therefore, the development of large-area, uniform, and scalable nanostructuring methods is essential. Techniques such as roll-to-roll nanoimprinting, nanosphere lithography, and colloidal self-assembly can enable the fabrication of plasmonic structures over large areas with high precision. Furthermore, research on hybrid plasmonic–dielectric structures can offer a balance between cost and performance.
(2) A major hurdle that hinders the large-scale deployment of PSCs is their limited stability. Plasmonic nanostructures, while effective in boosting optical absorption and electron extraction, can also introduce additional interfaces and materials that can compromise the device’s longevity. The degradation of perovskite materials under illumination, humidity, and temperature fluctuations is further exacerbated by the presence of plasmonic particles. Developing encapsulation techniques that effectively isolate the plasmonic–perovskite interface from environmental stressors is crucial. Researchers can also explore the use of more stable plasmonic materials or hybrid nanostructures that combine the benefits of multiple materials. Additionally, optimizing the perovskite composition and interface engineering can further improve the stability of PSCs.
(3) There are potentially detrimental aspects of plasmonic nanomaterials for the performance of solar cells. Although plasmonic materials have shown remarkable advantages in improving the performance of solar cells, they can also bring some harmful effects, including the introduction of more defects, an increase in recombination centers, the generation of hotspots, the induction of material degradation, and environmental pollution. To fully leverage their advantages while mitigating the harmful impacts, continuous technological research and innovation, as well as the optimization of production processes and environmental protection measures, are essential for achieving sustainable development. By optimizing the synthesis methods of nanomaterials, controlling plasma treatment parameters, and improving device structures, we can maximize the benefits of plasma nanomaterials while minimizing their adverse effects on solar cell performance.
(4) Most research on plasmonic-enhanced PSCs has focused on laboratory-scale devices with small active areas. As the device size increases, challenges arise due to non-uniform distribution of light, plasmonic hotspots, and increased resistance losses, resulting in a significant drop in the overall efficiency. Future research should focus on integrating plasmonic-enhanced PSCs into larger photovoltaic systems, considering factors such as module design, interconnection strategies, and overall system optimization. This includes developing methods to mitigate efficiency losses in large-area devices and optimizing the integration of PSCs with other photovoltaic technologies, such as silicon-based cells, in tandem or multi-junction configurations.
(5) We could consider integrating plasmonic technology with cutting-edge innovative technologies such as artificial intelligence (AI) and machine learning [154]. For instance, by leveraging big data analysis and machine learning algorithms, we can swiftly screen and predict potential novel materials in plasmonic technology [155]; with the assistance of AI’s optimization algorithms, we can fine-tune the plasmonic process parameters to achieve customized material properties [156]; AI’s formidable data processing capabilities enable it to explore material combinations and process conditions that are difficult to reach with traditional methods [157,158]. In this way, we can not only enhance material research and development efficiency, optimize material performance, and facilitate the discovery of new materials but also improve equipment performance, achieve intelligent control, and optimize process flows, thereby accelerating technological innovation, boosting industrial competitiveness, and promoting sustainable development.

In summary, the integration of plasmonic structures in PSCs represents a significant step forward in the pursuit of highly efficient and stable photovoltaic devices. With continued research and innovation, it is anticipated that plasmonics will play an increasingly important role in the transition towards sustainable and renewable energy sources, ultimately contributing to a greener and more sustainable future.

## Figures and Tables

**Table 1:** Summary of photovoltaic parameters for PSCs incorporating noble metal and their core–shell plasmonic nanomaterials. (The numbers in parentheses indicate the reference device performance.)

Materials	Size	Position of NPs	*J_SC_* (mA/cm^2^)	*V*_OC_ (V)	FF (%)	∆FF (%)	PCE (%)	∆PCE (%)	Ref
Ag nanoparticles	25 nm	ETL (TiO_2_)	18.91 (17.85)	0.90 (0.88)	70.20 (69.46)	1.07	11.96 (10.96)	9.12	[94]
Ag nanomaterials	10–100 nm	ETL	1.13 (1.05)	24.51 (24.67)	80.93 (75.13)	7.72	22.42 (19.52)	14.86	[113]
Ag NPLs	70 ± 20 nm	HTL (PEDOT: PSS)	15.40 (13.70)	0.92 (0.88)	68.20 (70.70)	−3.54	9.60 (8.50)	12.94	[95]
Ag NRs	20 nm (200 nm)	Perovskite	22.18 (21.08)	1.12 (1.12)	81.68 (78.36)	4.24	20.29 (18.50)	9.68	[96]
Ag nanoparticles	30 nm	ETL (TiO_2_)	13.14 (10.96)	0.78 (0.72)	60.18 (58.42)	3.01	6.15 (4.57)	34.57	[97]
Ag NCs	70 nm	ETL (PCBM)	21.40 (19.50)	1.00 (1.00)	62.00 (61.00)	1.64	13.30 (11.86)	12.14	[98]
Ag NPLs	79 ± 6 nm	Perovskite	24.41 (19.89)	0.85 (0.90)	65.00 (65.00)	0.00	13.46 (11.63)	15.74	[99]
Ag@TFP NPs	10–15 nm	Between perovskite and HTL	24.79 (23.52)	1.19 (1.15)	80.44 (78.96)	1.87	23.86 (21.54)	10.77	[114]
Ag@TiO_2_@Pa	28 nm	MAPbI_3_	21.69 (20.71)	1.13 (1.12)	83.00 (79.00)	5.06	20.24 (18.37)	10.18	[100]
Ag@TiO_2_	42 nm	ETL	22.00 (20.20)	1.06 (1.03)	69.00 (67.00)	2.99	16.30 (14.50)	12.41	[101]
Ag@SiO_2_	40 nm	ETL (TiO_2_)	23.04 (20.23)	1.02 (1.00)	62.17 (60.45)	2.85	14.61 (12.23)	19.46	[102]
Ag@SiO_2_	50 nm	HTL (PEDOT: PSS)	22.58 (20.21)	1.01 (0.97)	75.50 (74.40)	1.48	17.22 (14.58)	18.11	[115]
Au NRs	104.8 ± 6.8 nm (37.4 ± 1.8 nm)	Between perovskite and HTL	24.82 (22.88)	1.09 (1.08)	81.18 (80.47)	0.88	22.02 (19.96)	10.32	[64]
Au NRs	50 nm (20 nm)	Perovskite	21.60 (21.03)	1.10 (1.09)	81.92 (78.65)	4.16	19.46 (18.02)	7.99	[116]
Au NSs	40 nm	ETL(TiO_2_)	22.30 (20.97)	1.13 (1.11)	80.00 (76.00)	5.26	20.06 (17.64)	13.72	[103]
Au NRs	17 nm (93 nm)	Perovskite	23.72 (22.98)	1.11 (1.11)	82.47 (78.48)	5.08	21.73 (20.12)	8.00	[104]
Au nanoparticles	15 nm	HTL (Spiro-OMeTAD)	20.04 (19.63)	0.95 (0.96)	66.96 (67.23)	−0.40	12.74 (12.66)	0.63	[105]
Au NBs	45–50 nm (15–18 nm)	HTL(VOx)	22.68 (—)	1.08 (—)	77.10 (—)	—	18.84 (16.02)	17.60	[107]
Au NOs	115 nm	ETL(TiO_2_)	23.63 (22.29)	1.08 (1.05)	73.90 (72.38)	2.10	19.05 (16.95)	12.39	[108]
Au@SiO_2_ NPs	80 nm	ETL	16.91 (14.76)	1.02 (1.04)	64.00 (67.00)	−4.48	11.40 (10.70)	6.54	[109]
Au@SiO_2_ NPs	18 nm	ETL	22.30 (20.90)	1.07 (1.04)	72.12 (—)	—	19.42 (17.76)	9.35	[110]
Au@CdS NPs	35 nm	Between perovskite and HTL	23.14 (21.40)	1.12 (1.09)	79.90 (75.60)	5.69	20.67 (17.71)	16.71	[111]
Au@PSS NPs	54 nm	HTL (PEDOT: PSS)	23.34 (20.41)	1.06 (1.07)	70.50 (67.30)	4.75	16.53 (13.91)	18.84	[112]
Au@GO NPs	28 nm	Between perovskite and HTL (PEDOT: PSS)	18.56 (17.05)	1.02 (0.99)	74.00 (72.00)	2.78	14.00 (12.17)	15.04	[117]
Au@SiO_2_ NPs	−25 nm	HTL (NiO)	20.86 (20.40)	1.12 (1.12)	79.28 (79.72)	−0.55	18.52 (18.21)	1.70	[118]
Au@NiO NPs	−25 nm	HTL (NiO)	21.75 (20.40)	1.15 (1.12)	82.42 (79.72)	3.39	20.61 (18.21)	13.18	[118]
Au@SiO_2_ NPs	14 nm	ETL (between c-TiO_2_ and m-TiO_2_)	20.73 (19.86)	1.08 (1.08)	78.29 (75.71)	3.41	17.55 (16.18)	8.47	[119]
Au@TiO_2_ NPs	80 nm	Between porous TiO_2_ and MAPbI_3_	23.12 (17.40)	1.04 (0.98)	75.50 (73.70)	2.44	18.24 (12.59)	44.88	[120]
Au@TiO_2_ NPs	70 nm	Perovskite	22.93 (18.70)	0.97 (0.93)	83.30 (82.28)	1.24	18.47 (14.32)	28.98	[121]

**Table 2:** Summary of photovoltaic parameters for PSCs incorporating other plasmonic materials. (The numbers in parentheses indicate the reference device performance.)

Materials	Size	Position of NPs	*J_SC_* (mA/cm^2^)	*V*_OC_ (V)	FF (%)	∆FF (%)	PCE (%)	∆PCE (%)	Refs
Al NPs	20–70 nm	Between ETL (PCBM) and electrode	18.15 (16.71)	0.97 (0.96)	67.40 (65.10)	3.53	11.74 (10.54)	11.39	[126]
Au@Ag nanocuboids	15 nm	ETL(m-TiO_2_)	23.60 (20.68)	1.04 (1.00)	74.60 (73.00)	2.19	18.31 (15.16)	20.78	[132]
Au-Ag alloy popcorns	150 ± 50 nm	ETL(m-TiO_2_)	16.46 (15.51)	0.95 (0.92)	66.00 (63.00)	4.76	10.30 (8.90)	15.73	[133]
AuAg@AuAg core–shell alloy nanocrystals	46 nm	HTL (PEDOT: PSS)	21.89 (20.42)	1.02 (0.95)	77.20 (71.60)	7.82	16.76 (13.14)	27.55	[134]
AuAgNPrisms@SiO_2_	~40 nm	Between perovskite and HTL	7.08 (5.33)	0.97 (1.05)	67.00 (62.00)	8.06	4.60 (3.43)	34.11	[135]
Si NPs	140 nm	Between perovskite and ETL	22.40 (22.00)	1.06 (1.05)	78.90 (77.00)	2.47	18.80 (17.70)	6.21	[136]
Au@Ag@SiO_2_ core–shell nanocuboids	50 nm	Perovskite	23.76 (20.87)	1.05 (1.05)	71.39 (70.88)	0.72	17.78 (15.47)	14.93	[137]
SiO_2_@Ag@OC-TiO_2_ NW	400 nm	ETL(TiO_2_)	20.51 (19.36)	1.05 (1.00)	69.80 (64.50)	8.22	15.09 (12.54)	20.33	[138]
Au nanoparticles and MgO	40 nm (400 nm)	Between perovskite and ETL	21.76 (19.49)	1.09 (0.94)	68.00 (66.00)	3.03	16.10 (12.00)	34.17	[139]
Au-Ag nanoalloy	40 nm	ETL(TiO_2_)	21.75 (20.38)	1.02 (1.03)	65.70 (63.90)	2.82	14.42 (13.05)	10.50	[140]
Cu-Ag alloy NPs	200 nm	Between perovskite and electrode	22.96 (—)	1.12 (—)	73.20 (—)	—	18.89 (13.68)	38.08	[141]
AgOx@Ag NPs	40 nm	ETL(m-TiO_2_)	23.35 (21.86)	1.12 (1.09)	77.75 (75.00)	3.67	20.33 (17.87)	13.77	[142]
Au@PAT NPs	22 nm	MAPBI_3_	21.71 (21.20)	1.15 (1.12)	82.17 (78.00)	5.35	20.52 (18.59)	10.38	[143]
Au/Ag NSs	~60 nm	ETL(m-TiO_2_)	—	—	—	—	4.90 (3.90)	25.64	[144]
Submicron s-TiO_2_ NPs	160 nm	ETL(m-TiO_2_)	21.61 (20.28)	1.05 (1.08)	68.00 (67.00)	1.49	16.72 (16.31)	2.51	[145]
Au@TiO_2_ nanofibers	60 nm	ETL	21.63 (19.14)	0.99 (0.84)	70.00 (58.00)	20.69	14.92 (9.23)	61.65	[146]
Au nanorods@MgO	12 nm (40 nm)	ETL (c-TiO_2_)	22.35 (20.10)	1.04 (1.02)	75.00 (72.00)	4.17	17.40 (14.70)	18.37	[147]
Ge NPs	100 nm	ETL (m-TiO_2_)	22.93 (21.10)	1.06 (1.08)	75.91 (71.42)	6.29	18.59 (16.24)	14.47	[148]
GNR@SiO_2_	55 nm	Perovskite	24.25 (24.16)	1.08 (1.06)	81.41 (79.24)	2.74	23.26 (20.29)	14.64	[149]
SiO_2_@Ag@Ag_2_S NPs	120 nm	Perovskite	24.66 (22.17)	1.06 (1.06)	76.00 (74.30)	2.29	19.88 (17.49)	13.66	[150]
TiN NPs	20 nm	Perovskite	26.45 (25.09)	1.06 (1.05)	76.07 (72.70)	4.64	21.37 (19.07)	12.06	[151]
Au@SiO_2_@Graphene NPs	17 nm	CH_3_NH_3_PbI_3_	28.17 (21.20)	1.00 (0.98)	71.00 (68.00)	4.41	20.05 (14.21)	41.10	[152]
rGO NPs	—	ETL	22.51 (21.01)	1.06 (1.05)	72.00 (72.00)	0.00	17.08 (15.93)	7.22	[153]

## Data Availability

Data are contained within the article.
